# Protocol for Post-Mortem Micro-CT Imaging of Coronary Arteries in Low-Mass Neonatal Puppy Hearts Using Barium-Based Contrast

**DOI:** 10.3390/ani16111617

**Published:** 2026-05-26

**Authors:** Agata Godlewska, Olga Szaluś-Jordanow, Anna Jaśkiewicz, Jakub Jaroszewicz, Wojciech Święszkowski, Wojciech Mądry, Michał Buczyński, Karolina Barszcz

**Affiliations:** 1 Department of Morphological Sciences, Institute of Veterinary Medicine, Warsaw University of Life Sciences (SGGW), Nowoursynowska 159, 02-776 Warsaw, Poland; karolina_barszcz@sggw.edu.pl; 2 Department of Small Animal Diseases with Clinic, Institute of Veterinary Medicine, Warsaw University of Life Sciences (SGGW), Nowoursynowska 159c, 02-776 Warsaw, Poland; olga_szalus-jordanow@sggw.edu.pl; 3 Auxilium 24-Hour Veterinary Clinic, Królewska 64, 05-822 Milanówek, Poland; chatanga@gmail.com; 4 Faculty of Materials Science and Engineering, Warsaw University of Technology, Wołoska 141, 02-507 Warsaw, Poland; jakub.jaroszewicz@pw.edu.pl (J.J.); wojciech.swieszkowski@pw.edu.pl (W.Ś.); 5 Department of Heart, Chest and Transplant Surgery, Medical University of Warsaw, Żwirki i Wigury 63A, 02-091 Warsaw, Poland; wojciech.madry@wum.edu.pl (W.M.); michal.buczynski2@wum.edu.pl (M.B.)

**Keywords:** micro-computed tomography (micro-CT), gelatin–barium sulfate (gelatin–BaSO_4_) contrast medium, agar embedding, vascular casting

## Abstract

**Aim:** We aimed to provide a structured ex vivo protocol for cardiopulmonary micro-CT that combines gelatin–barium sulfate (gelatin–BaSO_4_) contrast medium with agar embedding in neonatal canine cardiopulmonary specimens. **Materials and Methods:** Heart–lung specimens from 23 puppies that died shortly after birth were collected, stored at −20 °C, and then slowly thawed prior to imaging. Before perfusion, body mass and heart–lung complex mass were recorded. Body mass ranged from 140 to 951 g, and heart–lung complex mass ranged from 1.2 to 51.2 g. The cranial and caudal venae cavae, the brachiocephalic trunk, and the left subclavian artery were ligated. A catheter was introduced into the thoracic aorta. Contrast was prepared by dissolving porcine gelatin in hot water and mixing with a commercial BaSO_4_ suspension. The mixture was maintained at a warm temperature to remain free-flowing and was delivered at low pressure until uniform opacification of the coronary and pulmonary arteries was observed. After in situ gelation, the organs were embedded in warm agar and sealed to limit motion and dehydration. Scans were performed on a benchtop system (120 kV, ~83 µA, ~1200 projections, ~2 s exposures; voxel ~40 µm). Reconstruction was performed in XMReconstructor, with post-processing in Falcon and RadiAnt. The reconstructed micro-CT datasets were reviewed anatomically by a medical cardiologist and a veterinary cardiologist, whereas vascular filling was evaluated semi-quantitatively by three observers with expertise in veterinary anatomy and cardiology. **Results:** In all specimens examined, the main coronary artery course was assessable. **Conclusions:** The gelatin–BaSO_4_ contrast medium combined with agar immobilization provides a simple, lead-free, and affordable approach for structured cardiopulmonary micro-CT in very small post-mortem specimens. In the examined specimens, the workflow provided visually consistent low-pressure vascular opacification without gross evidence of vessel rupture or motion-related acquisition failure under the conditions of this study. Practical mitigations included temperature/viscosity control, avoidance of phosphate buffers, container sealing, and minimization of particle aggregation, bubbles, and dehydration. The protocol may complement conventional autopsy in very small post-mortem specimens in similar ex vivo research settings.

## Simple Summary

Conventional post-mortem examination remains the basis for diagnosing congenital cardiopulmonary abnormalities, but in extremely small specimens, its diagnostic value may be limited by organ size, tissue fragility, and the irreversible nature of dissection. Micro-computed tomography (micro-CT) provides a non-destructive means of visualizing these structures in two-dimensional multiplanar reconstructions and three-dimensional renderings before further anatomical sectioning. With contrast enhancement and careful immobilization, micro-CT has become a standard approach for studying low-mass hearts in both human and mouse specimens. In this study, we describe a practical ex vivo workflow for post-mortem micro-CT of the heart and lungs in puppies that died shortly after birth. The protocol is based on gentle arterial filling with a warm gelatin–barium sulfate contrast medium, followed by immobilization of the combined heart–lung in agar. We provide not only the basic formulation and scanner settings but also procedural details, including the vessel ligation strategy, catheter placement, temperature control of the contrast mixture, visual signs of satisfactory vascular filling, and measures to prevent leakage, dehydration, bubble formation, and contrast aggregation. The method uses affordable and widely available materials and preserves the tissue for subsequent conventional autopsy and other post-mortem analyses. Although the filling procedure is time-consuming and requires careful manual handling, it enabled consistent visualization of the main coronary arteries and pulmonary vasculature in the examined very low-mass specimens. The protocol may be useful for veterinary anatomists, pathologists, cardiologists, and researchers working with neonatal, fetal, or other very small cardiopulmonary specimens in which conventional gross examination alone may be insufficient.

## 1. Introduction

Micro-CT is a high-resolution X-ray technique that acquires 2D projections and reconstructs high-resolution isotropic voxel-based 3D volumes. In veterinary and experimental settings, it is used ex vivo primarily for non-destructive visualization of internal anatomy, achieving micrometer-scale isotropic voxels in small specimens, including fetal/neonatal material [1]. In such small organs, conventional dissection is inherently limited. Once cuts are made, alternative planes are lost. Therefore, micro-CT provides a faithful three-dimensional anatomical framework that can guide, or in selected cases partially replace, conventional sectioning [2,3,4]. Although in vivo small animal micro-CT is feasible, it operates at lower resolution under strict dose and motion constraints. Even with standardized, few-minute, low-dose protocols that make acquisitions practical, it remains primarily a preclinical research tool rather than a routine diagnostic exam [2,5]. Beyond absorption-based imaging, phase-contrast micro-CT can enhance the definition of soft-tissue edges and the visibility of delicate structures. Still, it typically relies on specialized setups and is not yet routine in veterinary workflows [2,5].

A major limitation of micro-CT is its poor intrinsic soft-tissue contrast [2]. A notable exception is the lung, where the natural air–tissue interface provides strong inherent contrast [5]. For other soft tissues, workflows routinely use exogenous contrast media, which are broadly categorized into diffusible agents and vascular lumen fills [2,6,7,8]. Within the diffusible class, Lugol’s iodine (I_2_KI) and iodine-in-ethanol (I_2_E) solutions penetrate fixed tissue rapidly and provide versatile enhancement of embryonic structures [2,3,4,6,8]. Heteropoly acids, especially phosphotungstic acid (PTA) and phosphomolybdic acid (PMA), diffuse more slowly [8]. PTA demonstrates relative collagen affinity, providing contrast complementary to iodine and enabling the selective depiction of vessel walls and fibrous matrices [3,6,7]. Additionally, gadolinium chelates have been used ex vivo in dual-energy or complementary staining workflows [9].

The second group, vascular casting media, fills the lumen to generate high-fidelity 3D reconstructions of the vasculature. Silicone-based resins, such as Microfil MV-122, polymerize in situ to form durable casts but often require higher perfusion pressures and can introduce artifacts, including rounded edges or vessel distension [10,11]. Barium-based perfusates, on the other hand, provide a lead-free alternative, allowing lower perfusion pressures with cerebrovascular visualization equal to or superior to that of silicone resins [10,12,13].

Achieving micrometer-scale resolution requires long per-projection exposures and many views, so benchtop micro-CT scans are typically hour-scale and accrue a non-negligible radiation dose. Specimens must therefore be rigidly immobilized and protected from dehydration to prevent motion- and shrinkage-related artifacts during acquisition [2,5,6,12]. Immobilization options include agar/agarose and low-melt gelatin, alginate, or mechanical jigs/3D-printed holders. Sealing the container to limit evaporation is recommended for long scans [3,12].

In very small neonatal specimens, post-mortem evaluation is further complicated by the fact that many diagnostically relevant structures are not only very small but also spatially complex. This applies particularly to the coronary arteries, outflow tracts, aortic arches, and pulmonary vessels, whose interpretation may depend on preserved three-dimensional relationships rather than on isolated inspection of individual structures. Once conventional dissection is initiated, these native relationships are progressively altered and cannot be reconstructed in their original form [4,14,15]. For this reason, micro-CT may serve not only as a diagnostic adjunct but also as a method of permanent anatomical documentation, allowing repeated review of the same specimen by different observers and facilitating subsequent correlation with gross pathology, histology, and teaching material [14,15,16]. This advantage is particularly relevant in rare congenital malformations, where each post-mortem specimen may provide unique morphological information. A protocol-oriented approach is particularly important in this context because the quality of ex vivo vascular micro-CT depends not only on scanner settings but also on specimen handling, perfusion route, contrast temperature and viscosity, prevention of leakage, and stabilization during long acquisitions. Small deviations at any of these steps may affect vascular opacification and image interpretability [3,10,12,17]. For this reason, detailed methodological reporting is essential if cardiopulmonary micro-CT is to become a transferable methodological approach rather than a center-specific technical exercise [15,16,18].

In addition, interpretation of complex congenital malformations may depend not only on the morphology of individual cardiac structures but also on the preservation of spatial relationships among the heart, the great vessels, the ductus arteriosus, and the lungs. This is particularly relevant in anomalies of the outflow tracts and aortic arch, where accurate anatomical interpretation may require assessment of the entire cardiopulmonary complex rather than the isolated heart [4,13,19].

This article presents a structured protocol for contrast administration and specimen stabilization in cardiopulmonary micro-CT and justifies key methodological choices based on the authors’ experience with neonatal/fetal canine and feline material. The primary contribution of the study is methodological, namely the description of a practical ex vivo workflow for low-pressure vascular filling and stabilization of the intact heart–lung complex in very low-mass post-mortem specimens, rather than the generation of novel biological or diagnostic findings.

## 2. Materials and Methods

### 2.1. Specimens and Pre-Imaging Handling

Preparation and all procedural steps were adapted from protocols described previously by Szaluś-Jordanow et al. [13,19]. Cardiopulmonary specimens, comprising the heart and lungs, were collected during post-mortem examination from 23 puppies (16 females and 7 males) that died shortly after birth. No procedures were performed on live animals.

Immediately after collection, the specimens were stored frozen at −20 °C for approximately 1 to 3 months until imaging. Before contrast administration, each specimen was thawed slowly to reduce the risk of tissue cracking, abrupt fluid release, and mechanical damage to delicate vessels. Body mass ranged from 140 to 951 g (median 276.3 g, IQR 226–460 g), whereas heart–lung complex mass ranged from 1.2 to 51.2 g (median 9.2 g, IQR 4.5–15.1 g), corresponding to a median of 3.4% of body mass (IQR 1.9–4.8%).

Before perfusion, each specimen was visually inspected to assess overall integrity and suitability for imaging. Particular attention was paid to the continuity of the thoracic aorta, the condition of the atrial and venous inflow regions, and the preservation of the pulmonary hilus.

### 2.2. Vessel Ligation and Preparation for Arterial Perfusion

**Figure 1:**
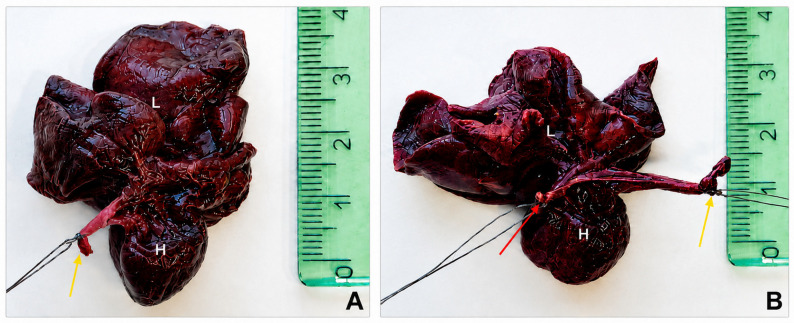
(**A**,**B**) Gross anatomical specimen showing the heart (H), approximately 1 cm in size, in continuity with the lungs (L). The ligation sites of the venae cavae are indicated by arrows: yellow arrow—cranial vena cava; red arrow—caudal vena cava.

**Figure 2:**
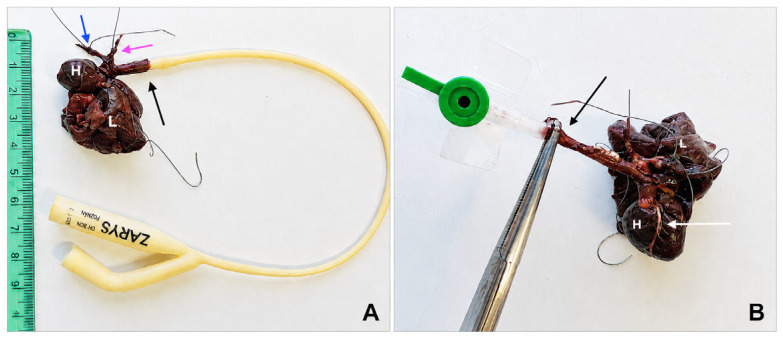
Gross anatomical specimen showing the heart (H), in continuity with the lungs (L). (**A**) The blue arrow indicates the ligated brachiocephalic trunk, representing the first branch of the aortic arch in the dog. The pink arrow indicates the ligated left subclavian artery, representing the second branch of the aortic arch. The black arrow indicates the thoracic aorta, into which a Foley catheter with an inflated balloon was inserted to occlude the lumen and enable contrast administration. (**B**) An alternative method of contrast administration is shown. An intravenous catheter is inserted into the thoracic aorta (black arrow) and secured using hemostatic forceps to prevent leakage. The initial portion of the contrast medium is visible within the paraconal interventricular branch (white arrow).

To achieve selective intravascular retention of the contrast medium and minimize uncontrolled outflow, the major venous and arterial branches that could act as escape pathways were ligated. Specifically, both the cranial and caudal venae cavae, as well as the brachiocephalic trunk and the left subclavian artery (Figure 1 and Figure 2A). These ligatures were placed using fine suture material under direct gross visualization. The ligation strategy was designed to direct the injected contrast medium preferentially into the ascending aorta, coronary, and pulmonary vessels while minimizing reflux or external leakage. In very small specimens, even minor traction can change the natural orientation of the great vessels.

### 2.3. Catheter Placement

After ligation of the selected vessels, arterial access was established through the thoracic aorta. Depending on specimen size and vessel diameter, either an 8 Fr/Ch Foley catheter (ZARYS International sp. z o.o., Zabrze, Poland) or a small intravenous catheter (Becton, Dickinson and Company, Franklin Lakes, NJ, USA) was used (Figure 2). In the case of a Foley catheter, the balloon could help stabilize the catheter within the aortic lumen and reduce backflow. In smaller specimens, an intravenous catheter secured in place with a clamp or hemostatic forceps provided a practical alternative when the aortic diameter did not allow secure placement of a Foley catheter.

Correct catheter positioning was important for two reasons. First, the catheter tip had to remain stable during the relatively prolonged manual injection. Second, the catheter could not be advanced in a manner that would obstruct the aortic root or mechanically compromise the proximal coronary outflow. Once satisfactory positioning had been achieved, the specimen was oriented so the operator could observe the heart’s surface directly during injection and assess progressive filling of the subepicardial coronary vessels.

### 2.4. Preparation of the Gelatin–BaSO_4_ Contrast Medium

The contrast medium was prepared immediately before use to ensure adequate fluidity and homogeneous suspension of the radiopaque component. A gelatin-based carrier was selected because it remains injectable at body temperature and subsequently gels on cooling, thereby helping to maintain intraluminal distribution after perfusion. The radiopaque phase consisted of a commercially available BaSO_4_ suspension (Barium sulfuricum Medana, 1 g/mL; POLPHARMA Pharmaceutical Works S.A., Medana Branch, Sieradz, Poland), chosen because barium sulfate provides high X-ray attenuation, is lead-free, and is readily available.

The gelatin base was prepared by dissolving 8 g of porcine gelatin powder in 50 mL of water heated to 90–95 °C with continuous stirring until a uniform solution was obtained. The mixture was not used until the gelatin had fully dissolved and no visible clumps remained. The gelatin solution was combined with 25 mL of BaSO_4_ suspension, and the mixture was stirred until homogeneous. Because the viscosity of gelatin-based mixtures increases rapidly as temperature decreases, the final contrast medium was maintained at 37–40 °C in a water bath until administration.

In practice, contrast preparation required attention to both ingredient proportions and the mixture’s physical behavior. If the medium cooled prematurely, viscosity increased, and injection became more difficult, particularly in the smallest specimens. Conversely, insufficient homogenization could lead to particle aggregation and inconsistent opacification. Therefore, the mixture was stirred until visually uniform and kept warm until injection.

### 2.5. Contrast Administration

**Figure 3:**
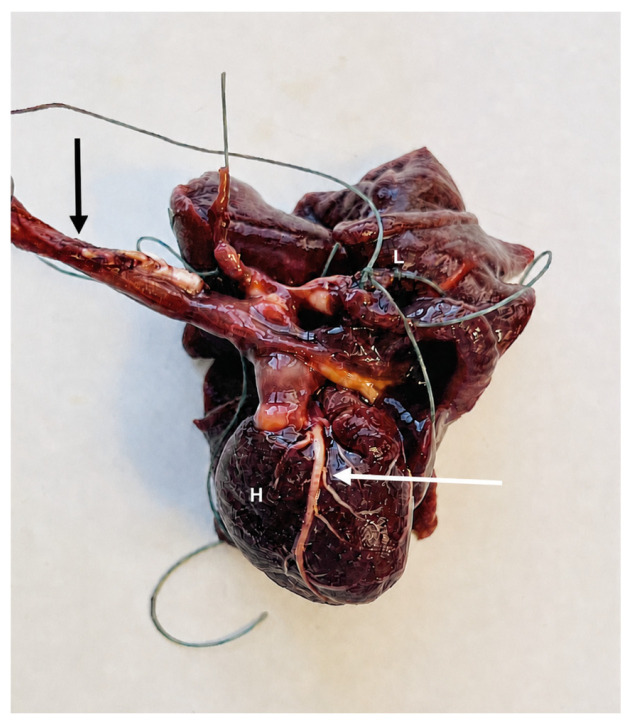
Gross anatomical specimen showing the auricular surface of the heart (H) and lungs (L) after contrast administration. The black arrow indicates the descending aorta. The white arrow indicates the paraconal interventricular branch and its collateral branches. The cardiac chambers are uniformly distended, consistent with adequate intravascular filling during contrast injection.

The warmed gelatin–BaSO_4_ contrast medium was injected slowly into the thoracic aorta under gentle manual pressure and continuous visual monitoring of the specimen surface (Figure 3). Because the neonatal coronary vasculature is extremely delicate, excessive pressure may have caused vessel overdistension, rupture, or non-physiological filling patterns.

During injection, the operator monitored visual signs of satisfactory perfusion. These included gradual opacification and distension of the major subepicardial coronary branches, visible enhancement of collateral branches on the cardiac surface, and filling of the pulmonary vasculature. Uniform but not excessive chamber distension was regarded as a favorable sign of central vascular filling without obvious overpressurization. Injection was continued until the coronary and pulmonary vessels were macroscopically visible.

Once satisfactory perfusion was achieved, the catheter was carefully removed to avoid traction injury at the injection site. The aorta was then ligated to reduce backflow of the contrast medium. Because the gelatin carrier solidifies upon cooling, the specimen was subsequently left undisturbed for a period sufficient to allow in situ gelation and stabilization of the intravascular contrast column.

In practice, injection was adjusted continuously according to the resistance encountered in each specimen. If resistance increased abruptly, the procedure was temporarily paused, and the catheter position, vessel patency, and temperature of the contrast medium were reassessed. Particular attention was paid to signs of leakage around the catheter insertion site and to asymmetrical or unexpectedly poor filling of the visible subepicardial vessels. This cautious approach was especially important in the smallest specimens, where even slight overpressure could compromise vascular integrity or lead to non-uniform opacification.

### 2.6. Agar Embedding and Specimen Stabilization

To immobilize the organs during micro-CT acquisition, the heart–lung complex was embedded in agar. This step served two purposes: stabilizing the specimen to prevent motion artifacts and providing partial protection against dehydration during scanning. The agar solution was prepared by dissolving 8 g of agar powder in 100 mL of boiling water. Once the solution was cooled to approximately 40 °C, the specimen was fully immersed and left undisturbed until the gel solidified, ensuring stable positioning throughout the micro-CT acquisition. The container was sealed to minimize evaporation during scanning.

During embedding, care was taken to preserve the natural orientation of the heart and lungs to avoid compressing or rotating the specimen within the container. This was important not only for stabilization but also for subsequent anatomical interpretation, because artificial displacement of the cardiac base or pulmonary lobes could complicate assessment of spatial cardiopulmonary relationships.

### 2.7. Micro-CT Acquisition

All scans were performed using an Xradia XCT-400 system (Carl Zeiss Microscopy GmbH, Jena, Germany). Imaging was conducted at 120 kV and approximately 83 µA. For each specimen, 1200 projections were acquired over a full 360° rotation, with an exposure time of 2 s per projection. A 0.5× large-field-of-view objective was used, resulting in an isotropic voxel size of 40 µm × 40 µm × 40 µm.

### 2.8. Image Reconstruction and Post-Processing

Raw projection data were reconstructed using the XMReconstructor software, version 8.1, supplied with the scanner, using a filtered back-projection algorithm. No additional beam-hardening or ring-artifact correction beyond the default XMReconstructor reconstruction settings was manually applied. Following reconstruction, the datasets were reviewed and post-processed in Falcon Mx26 software (iCat Solutions Ltd., Norwich, UK) and RadiAnt DICOM Viewer, version 2025.2 (Medixant, Poznań, Poland). Multiplanar assessment and three-dimensional visualization were both used during interpretation, because coronary artery assessment benefits from the complementary strengths of axial, sagittal, and coronal planes, as well as from volume- and surface-rendered reconstructions.

Image interpretation followed the cardiac segmental analysis methodology described by Szaluś-Jordanow et al., which includes assessment of chamber morphology, septal continuity, outflow tract configuration, and coronary artery origins [13,19].

In addition to descriptive anatomical assessment, vascular filling was evaluated semi-quantitatively by three observers: two with expertise in veterinary anatomy and one with experience in veterinary cardiology. A simple three-point grading system was used: grade 0, non-diagnostic or insufficient vascular filling; grade 1, partial filling allowing identification of the main proximal coronary branches but limiting distal assessment; and grade 2, complete or near-complete filling allowing assessment of the main subepicardial coronary course and proximal branches. Any discrepancies were resolved by consensus. Because the study was designed as a methodological protocol rather than a validation study, formal statistical analysis of interobserver agreement was not performed.

## 3. Results

Across the specimens examined, the main coronary branches were consistently identifiable, regardless of whether they were normally developed or morphologically abnormal. The technique enabled clear delineation of the course of the subepicardial coronary arteries. Semi-quantitative assessment of vascular filling confirmed that all 23 specimens were suitable for evaluation of the main coronary artery course. 20/23 examinations were classified as grade 2, indicating complete or near-complete filling of the main subepicardial coronary branches, whereas 3/23 specimens were classified as grade 1 because of less uniform distal opacification but remained interpretable at the level of the main coronary vessels. No specimen was classified as grade 0. Similarly, concomitant pulmonary vascular opacification was observed in all examined heart–lung complexes, although the degree of distal pulmonary vessel filling varied between specimens.

In three specimens, the normal coronary ostia could not be identified at their expected sites in the aortic root. Before this finding was interpreted in the context of associated structural abnormalities, the datasets were reassessed in multiple reconstruction planes using three-dimensional volume- and surface-rendered views focused on the aortic root and proximal coronary course. These additional assessments did not reveal normally positioned coronary origins. The absence of visible coronary ostia in these cases was therefore interpreted in relation to the associated congenital structural defects rather than as a primary technical failure of the imaging protocol.

**Figure 4:**
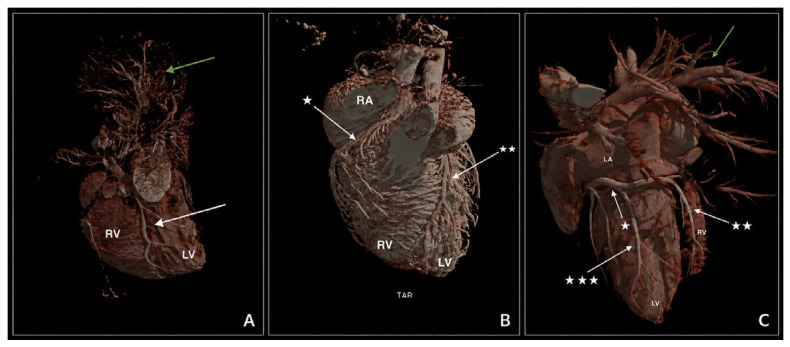
Micro-CT image obtained after contrast administration. RA—right atrium; LA—left atrium; RV—right ventricle; LV—left ventricle. (**A**) Auricular view of the heart and pulmonary vasculature. The green arrow indicates contrast-filled pulmonary vessels. The white arrow indicates the paraconal interventricular branch of the left coronary artery, within the paraconal interventricular groove. The image is presented in the same projection as the corresponding gross anatomical specimen in Figure 3. (**B**) Cardiac chambers and coronary vasculature. ★—right coronary artery; ★★—paraconal interventricular branch of the left coronary artery. (**C**) Cardiac chambers and major vessels. ★—left circumflex branch; ★★—subsinuosal interventricular branch; ★★★—left ventricular marginal branch. The green arrow indicates contrast-filled pulmonary vessels.

Overall, the applied workflow enabled consistent post-mortem visualization of coronary artery anatomy in small neonatal hearts, including both normal and pathologically altered vascular patterns. Figure 4 presents micro-CT reconstructions obtained from three different animals, illustrating the coronary arterial patterns following contrast administration. Although minor inter-individual differences in contrast distribution and soft tissue visualization are apparent, consistent and detailed delineation of the coronary arteries was achieved in all examined specimens. These findings indicate that, despite variability in contrast filling and tissue attenuation, the applied imaging protocol enables reliable visualization of the coronary arterial system across different individuals.

From a technical standpoint, the protocol proved feasible across a broad range of neonatal body and organ masses. Although minor variation was observed in the apparent degree of chamber filling, contrast distribution, and background soft-tissue attenuation, these differences did not prevent assessment of the main coronary branches. In practical terms, successful examinations were characterized by a distinct visualization of the subepicardial coronary course and concomitant opacification of the pulmonary vasculature. In contrast, suboptimal filling tended to affect only the most distal vascular detail rather than the overall interpretability of the specimen. The embedding procedure provided stable positioning throughout scanning, and no gross motion-related acquisition failures were observed.

## 4. Discussion

Micro-CT was used to evaluate coronary artery anatomy in neonatal dogs that died shortly after birth. Due to the small size of the hearts, which weigh only a few to several grams, the high spatial resolution of micro-CT enabled detailed visualization of the coronary vessels along their subepicardial course. Consistent visualization of the coronary arteries across specimens should be interpreted primarily as an indicator of the protocol’s technical feasibility rather than as a novel biological finding. The practical novelty of the present workflow lies in combining low-pressure administration of a gelatin–BaSO_4_ contrast medium with agar immobilization of the intact heart–lung complex in very low-mass neonatal specimens, thereby preserving cardiopulmonary spatial relationships during prolonged ex vivo micro-CT acquisition.

A central methodological decision concerns the choice of contrast. Iodine immersion protocols are well established for soft-tissue enhancement. However, they typically require formalin fixation and prolonged staining periods of two to four days, which may slightly reduce tissue mass. In contrast, vascular filling with gelatin–BaSO_4_ contrast medium can be completed in less than an hour, is applicable to unfixed hearts and lungs, and preserves tissue for subsequent gross and histological examination. Moreover, coronary vessel visualization with diffusible soft-tissue staining is generally less detailed than that achieved with barium-enhanced vascular micro-CT [2,3,6,7,9]. However, the present study did not include a direct experimental comparison with iodine-based staining protocols or other contrast methods. The discussion of alternative approaches is therefore intended to contextualize the methodological choices made in the present workflow rather than to demonstrate comparative superiority. While freezing can introduce histological artifacts at the cellular level, available evidence and our experience indicate that it does not materially alter gross cardiac anatomy relevant to micro-CT interpretation [19].

Previous studies have shown that barium-based vascular contrast media can provide high radiopacity at relatively low perfusion pressures and may avoid some limitations associated with polymerizing silicone casts, such as vessel distension or rupture. Hong et al. demonstrated in the mouse cerebrovasculature that barium-based formulations can support detailed vascular visualization, although their behavior depends on particle size, suspension stability, and buffer composition [12]. In the present protocol, BaSO_4_ was used in a warm gelatin carrier, which provided a free-flowing medium during injection and subsequently gelled upon cooling. This combination was selected to support low-pressure intraluminal filling, limit backflow, and reduce leakage in fragile neonatal specimens. However, the present study did not directly compare gelatin–BaSO_4_ with Microfil, iodine-based staining, or other vascular contrast protocols. Therefore, these comparisons should be interpreted only as a methodological context for the chosen workflow, not as evidence of superiority.

Agar embedding is the second pillar of image quality in this protocol. Ex vivo platforms, such as the Xradia XCT-400, combine long per-projection exposures (≈2 s), a large number of projections (≈1200 over 360°), and high tube settings (120 kV, 83 µA), resulting in hour-long acquisitions and a cumulative X-ray dose incompatible with living subjects. By setting a supportive matrix around the heart–lung complex, agar eliminates micro-motions and buoyancy shifts that would otherwise accumulate as misregistration across hundreds to thousands of projections [12]. This is particularly important at 40 µm isotropic voxel resolution with two-second exposures, where even subtle drift can degrade fine structures such as hypoplastic pulmonary branches. Dehydration during long scans results in measurable shrinkage and contrast drift, particularly at tissue–air interfaces. Sealing and gel embedding help to maintain moisture and geometry throughout acquisition [3,12].

From a post-mortem morphological standpoint, this method fills an important gap between conventional autopsy and destructive dissection [4,14,15,16]. The present study was not designed to generate novel biological insight into congenital cardiac development, nor to assess diagnostic accuracy or validate the protocol against an established gold standard. Instead, its purpose was to establish a structured methodological framework for post-mortem visualization of coronary and cardiopulmonary anatomy in very small specimens. Accordingly, the proposed approach should be interpreted primarily as a post-mortem morphological and methodological tool rather than as a clinically validated diagnostic method. Gross examination remains indispensable, but it may be difficult to follow the complete course of the coronary arteries or appreciate subtle spatial relationships within a malformed neonatal heart after serial cuts have been made. Micro-CT performed before dissection preserves the original three-dimensional context and allows repeated evaluation of complex anatomy in arbitrary planes. This is especially valuable in cases of congenital malformations in which coronary anomalies may coexist with abnormal outflow tract development, interrupted or hypoplastic great vessels, and pulmonary vascular abnormalities. Beyond its methodological application, the method also provides a durable digital anatomical record that may support later morphological review, comparative analysis, and teaching [14,16]. This is particularly important in rare malformations, where the available material is limited, and preservation of morphological information is essential.

An important and, in our view, insufficiently emphasized feature of this workflow is the deliberate preservation and imaging of the heart–lung complex as a single unit, which maintains native three-dimensional cardiopulmonary relationships and minimizes distortion associated with organ separation [3]. In congenital disease, the lungs should not be regarded merely as adjacent structures, but as part of the same developmental and hemodynamic system as the heart. Whereas many published micro-CT protocols are designed for isolated organs and therefore provide limited whole-organ-system context, our approach enables simultaneous assessment of intracardiac malformations, pulmonary arterial filling, pulmonary and parenchymal hypoplasia, and the spatial relationships between the cardiac base and thoracic vascular structures [3]. This integrated perspective may be particularly valuable in complex congenital malformations, in which assessment of the heart alone may be insufficient, as accurate anatomical interpretation often depends on understanding how the great vessels extend beyond the cardiac base and how they relate spatially to the pulmonary arteries and lung parenchyma [20]. This is especially relevant in defects such as transposition of the great arteries or interrupted aortic arch, in which isolated heart examination may not fully reveal the extracardiac course or continuity of the great vessels [20]. By preserving native relationships among the ventricles, outflow tracts, aortic arch, pulmonary arteries, and lungs, the present protocol enhances confidence in the diagnosis of complex conotruncal and great-vessel anomalies. This may be particularly informative in brachycephalic breeds and in cases of hydrops fetalis, in which combined cardiopulmonary developmental abnormalities have already been documented [13,19]. Accordingly, the protocol is not limited to coronary artery imaging, but also supports a broader, system-level morphological evaluation of neonatal cardiopulmonary malformations.

Preservation of the intact cardiopulmonary complex is also important for coronary artery assessment. In congenital heart disease, especially in conotruncal anomalies, coronary artery origins may be abnormal or displaced, and their recognition depends on accurate identification of the aortic root and its three-dimensional orientation [13,19,21,22]. This point is illustrated by the three specimens in the present series in which normally positioned coronary ostia could not be identified despite reassessment of the aortic root in multiple reconstruction planes and three-dimensional views. In such cases, the absence of visible coronary ostia should be interpreted cautiously and in relation to the overall morphology of the aortic root and great vessels, rather than as an isolated imaging finding. When the aortic root is hypoplastic, malpositioned, or structurally abnormal, loss of the native anatomical context may hinder visualization of the coronary ostia and complicate interpretation of the proximal coronary course [13,19,21,22].

Although the present protocol was applied only to neonatal canine specimens, its technical principles are likely transferable to other very small post-mortem cardiopulmonary specimens, including kittens, fetal puppies, and potentially other small mammalian neonates. In such cases, the main procedural elements would remain unchanged: careful preservation of the heart–lung complex, low-pressure arterial administration of a warmed gelatin–BaSO_4_ contrast medium, and agar-based immobilization during scanning. However, practical modifications would be required depending on vessel diameter, tissue fragility, gestational age, and organ mass. In particular, catheter size, injection volume, injection resistance, and contrast viscosity need to be adjusted individually. Therefore, extrapolation to other species or developmental stages should be regarded as technically plausible but requires specimen-specific optimization.

An additional practical strength of this micro-CT workflow is its potential to improve the standardization of post-mortem cardiopulmonary assessment across observers and institutions. In very small neonatal specimens, conventional autopsy findings may depend heavily on operator experience, the dissection order, and the extent to which fragile structures remain intact during handling. By contrast, volumetric micro-CT datasets can be stored, reoriented, and re-evaluated repeatedly without further damaging the specimen, facilitating second-opinion review and retrospective comparison of rare malformations. This is particularly relevant in veterinary perinatal pathology, where case numbers are low and individual specimens may represent unique morphological constellations. In such settings, structured ex vivo imaging protocols may help reduce descriptive variability, support teaching and multidisciplinary consultation, and create comparable image archives for future studies. Importantly, the value of this approach is not limited to documenting overt malformations. It may also improve recognition of subtle but diagnostically important spatial relationships, such as the continuity of the aortic arch, the relative arrangement of the outflow tracts, or the proximal course of the coronary arteries. Thus, beyond its immediate diagnostic role, the present protocol may contribute to a more systematic and shareable framework for morphological assessment of rare neonatal cardiopulmonary defects.

## 5. Conclusions

This study presents a structured post-mortem micro-CT protocol for imaging coronary arteries and cardiopulmonary structures in low-mass neonatal canine specimens. The use of a gelatin–BaSO_4_ contrast medium combined with agar embedding provided stable imaging conditions and consistent visualization of the coronary arteries in the examined specimens. Preservation of the intact heart–lung complex represents a key strength of the approach, facilitating anatomical interpretation of complex cardiac and great vessel relationships in very small post-mortem specimens. In addition to supporting detailed anatomical assessment, the method preserves the specimen for further examination and generates permanent three-dimensional datasets that may be useful for research, teaching, and comparative post-mortem analysis. The protocol may serve as a practical methodological complement to conventional autopsy in very small post-mortem cardiopulmonary specimens. The method may also facilitate retrospective case reviews and creation of reference image archives for rare neonatal malformations in very small post-mortem specimens of a similar type.

## 6. Limitations

Several limitations of the present study should be acknowledged. First, although the applied gelatin–BaSO_4_ contrast medium protocol consistently opacified the coronary arteries and pulmonary vasculature, complete filling of the most distal microvascular branches cannot be guaranteed. The technique primarily reflects lumen patency under low-pressure conditions rather than physiological perfusion and may therefore underestimate the extent of the smallest-caliber vessels.

Second, despite the generally reliable visualization of coronary arteries observed in this study, identification of coronary ostia may be challenging in cases of severe congenital malformations affecting the aortic root and great vessels. In such conditions, the apparent absence of proximal coronary segments may reflect altered spatial relationships or abnormal vessel origin rather than a true absence of coronary arteries. This limitation is inherent to post-mortem imaging techniques that depend on correct anatomical orientation.

Third, the study was performed on ex vivo specimens that had been previously frozen and thawed. Although this approach did not appear to affect gross anatomical interpretation or vascular filling, subtle microstructural alterations at the tissue level cannot be excluded. However, these changes are unlikely to influence the primary objective of three-dimensional vascular visualization.

Fourth, the manual nature of contrast administration represents an additional limitation. Although low-pressure injections were deliberately used to avoid vessel damage, the procedure is operator-dependent. It may introduce variability in contrast distribution, particularly in very small or structurally abnormal specimens.

Fifth, although a simple semi-quantitative grading of vascular filling was conducted to summarize technical interpretability, no formal quantitative image-quality metrics, statistical comparison between specimens, or formal interobserver agreement analysis were performed. In addition, the study was not designed to quantitatively compare vascular filling performance, distal vessel opacification, or image quality against alternative contrast media or imaging protocols.

Sixth, the generalizability of the findings is limited because the protocol was applied only to very low-mass neonatal canine specimens. Extrapolation to other species, age groups, organ sizes, or clinical imaging settings should therefore be made with caution.

Finally, no formal validation against an established gold standard was performed, and diagnostic accuracy was not assessed. Therefore, the present study should be interpreted as demonstrating technical feasibility and potential usefulness for post-mortem morphological evaluation in very small specimens, rather than establishing the clinical performance of the method.

## Data Availability

The datasets generated and/or analyzed during the current study, including additional post-mortem images and computed tomography reconstructions, are available from the corresponding author on reasonable request.

## Abbreviations

The following abbreviations are used in this manuscript:

micro-CT	micro-computed tomography
BaSO_4_	barium sulfate
BaCl_2_	barium chloride
I_2_KI	Lugol’s iodine
I_2_E	iodine in ethanol
PTA	phosphotungstic acid
PMA	phosphomolybdic acid
PBS	phosphate-buffered saline
IQR	interquartile range
